# The risk of thrombo-embolic events is increased in patients with germ-cell tumours and can be predicted by serum lactate dehydrogenase and body surface area

**DOI:** 10.1038/sj.bjc.6602791

**Published:** 2005-10-04

**Authors:** A-C Piketty, A Fléchon, A Laplanche, E Nouyrigat, J-P Droz, C Théodore, K Fizazi

**Affiliations:** 1 Institut Gustave Roussy, Villejuif, France; 2 Centre Léon Bérard, Lyon, France

**Keywords:** cancer of the testis, chemotherapy, cisplatin, germ-cell tumour, thrombosis

## Abstract

The aim of this study was to evaluate the risk of thrombo-embolic events (TEE) in patients with germ-cell tumours (GCT) who receive cisplatin-based chemotherapy, to compare this risk to that of a matched control group of non-GCT cancer patients, and to identify risk factors of TEE. The rate of TEE during the 6 months following the initiation of chemotherapy was assessed in 100 consecutive patients with GCT and in 100 controls with various neoplasms who were matched on sex and age, and who received first-line cisplatin-based chemotherapy during the same period of time at Institut Gustave Roussy, Villejuif, France. Data were subsequently tested on a validation group of 77 GCT patients treated in Lyon, France. A total of 19 patients (19%) (95% confidence interval (CI): 13–28) and six patients (6%) (95% CI: 3–13) had a TEE in the GCT group and the non-GCT control group, respectively (relative risk (RR): 3.4; *P*<0.01). Three patients from the GCT group died of pulmonary embolism. In multivariate analysis, two factors had independent predictive value for TEE: a high body surface area (>1.9 m^2^) (RR: 5 (1.8–13.9)) and an elevated serum lactate dehydrogenase (LDH) (RR: 6.4 (2.3–18.2)). Patients with no risk factor (*n*=26) and those with at least one risk factor (*n*=71) had a probability of having a TEE of 4% (95% CI: 1–19) and 26% (95% CI: 17–37), respectively. In the GCT validation set, 10 (13%) patients had a TEE; patients with no risk factor and those with at least one risk factor had a probability of having a TEE of 0 and 17% (95% CI: 10–29), respectively. Patients with GCT are at a higher risk for TEE than patients with non-GCT cancer while on cisplatin-based chemotherapy. This risk can be accurately predicted by serum LDH and body surface area. This predictive index may help to study prospectively the impact of thromboprophylaxis in GCT patients.

The integration of cisplatin-based chemotherapy and surgery in the first-line treatment of germ-cell tumours (GCT) has resulted in cure rates of approximately 80% in disseminated cases and more than 98% when the disease is localised to the testis (Culine *et al*, 1996; Bosl and Motzer, 1997). The emphasis of clinical trials in the last 15 years has been to reduce the amount of early and late toxicity in patients with good-risk GCT. Late complications mainly include secondary malignancies, bleomycin-induced pulmonary fibrosis, Raynaud's phenomenon, peripheral neurotoxicity, infertility, and psychological disorders (Fizazi *et al*, 2002a). Moreover, some authors have suggested that there is both an increased incidence of hypercholesterolaemia (Raghavan *et al*, 1992) and an increased cardiovascular risk although this is still a subject of debate (Doll *et al*, 1986; Nichols *et al*, 1992; Gerl, 1994; Huddart *et al*, 2003).

Even though the matter of arterial events in GCT survivors remains controversial, there have been isolated reports on the occurrence of venous thrombo-embolic events (TEE) in these patients. However, only a few studies have specifically focused on this subject (Cantwell *et al*, 1988; Weijl *et al*, 2000). It was originally described by Trousseau that patients with cancer are at increased risk for venous TEE, although the mechanisms are not fully elucidated (Trousseau, 1865). These mechanisms may include procoagulant factors released by tumour cells, tumour-mediated vascular compression, and secondary immobilisation (Levine *et al*, 2002). Whether or not the use of chemotherapy increases the thrombo-embolic risk in cancer patients is still a topic of debate (Gerl, 1994). The occurrence of acute TEE in young men receiving chemotherapy for GCT has raised concerns since these complications may be life threatening and may also be responsible for long-term complications.

However, there is no definitive evidence that the TEE risk is increased in patients with GCT compared to other cancer patients. Therefore, the objectives of this study were to assess the incidence of TEE in a series of patients with GCT, to compare this incidence to that of a control population of cancer patients, and to determine predictive factors for TEE.

## PATIENTS AND METHODS

### Patients

We retrospectively reviewed the incidence of TEE in a population of 100 consecutive patients with GCT, selected on the following criteria: male patients, age above 16 years, pathologic evidence of GCT (seminoma or non-seminoma), a testicular or an extragonadal primary site and first-line cisplatin-based chemotherapy given at Institut Gustave Roussy, Villejuif, France, from January 1992 to December 1998. Patients with previous chemotherapy or radiotherapy were excluded, as well as patients with a TEE diagnosed before the initiation of chemotherapy.

Thrombo-embolic event was defined as any venous or arterial thrombo-embolic complication (including superficial thrombophlebitis and thrombophlebitis on venous catheter) occurring from the first day of chemotherapy to 6 months later. This period was chosen because cisplatin has been previously associated with the occurrence of TEE and because this drug has a long-lasting activity (Schilsky *et al*, 1982; Gerl and Schierl, 2000). Radiologic evidence was required to ascertain the diagnosis of TEE and it was obtained by Doppler ultrasonography, CT scan, arteriography, or phlebography. The evolution of TEE was extensively studied and reported with respect to complications, relapse, and long-term functional sequel.

The incidence of TEE was also assessed in a control population and compared to that observed in patients with GCT. The control group consisted of 100 consecutive patients who were matched on the following criteria: sex, age, pathologic evidence of non-GCT neoplasm, and first-line cisplatin-based chemotherapy received at Institut Gustave Roussy during the same period of time. This control group was taken from an electronic database.

### Statistical methods

Patient characteristics were obtained from the charts; they are expressed as percentages or medians (range). The predictive value of initial characteristics on the occurrence of a TEE was assessed using the log-rank test (Peto *et al*, 1977) for univariate analysis, and the Cox's proportional hazards regression model (Cox, 1972) for multivariate analysis. Factors with predictive value were combined to build a predictive model. All tests were two sided. The model was tested on a separate set of 70 patients with GCT treated at the Centre Léon Bérard, Lyon, France during the same period of time (validation set).

## RESULTS

Characteristics from the study set of patients with GCT and the control group of patients with non-GCT malignancies are summarised in Table 1. Both groups received cisplatin-based chemotherapy, most often at a dose of 100 mg m^−2^ cycle^−1^ (Table 2). Patients from the control group had a large range of malignancies and therefore they received a variety of different chemotherapy regimens. However, all of them received cisplatin-based chemotherapy; 31% of them received the rapidly recycled cisplatin, doxorubicin, and ifosfamide (API-AI) regimen (Fizazi *et al*, 1997). Patients from the GCT group received cisplatin and etoposide with or without bleomycin (BEP or EP) (Culine *et al*, 2003), or cisplatin, doxorubicin, cyclophosphamide alternated with vinblastine and bleomycin (CISCA-VB regimen) (Fizazi *et al*, 2002b) (Table 2). Allocation of patients with disseminated GCT to the IGCCCG prognostic classification (IGCCCG, 1997) was as follows: good-risk group: 66%; intermediate group: 4%; and poor-risk group: 30%. Besides tumour origin and chemotherapy, the two groups were comparable (Table 1). However, patients from the GCT group were treated less often via a central catheter than patients from the non-GCT control group (43 *vs* 74%, *P*=0.001). Criteria for selecting patients for a central venous catheter included mostly a poor peripheral venous access at examination and the planned duration of chemotherapy (typically a central vein catheter was recommended in patients planned to receive more than three cycles of chemotherapy). Patients from the GCT group also had more often a disseminated disease than those from the non-GCT control group (82 *vs* 57%, *P*=0.001). However, this is balanced by the fact that patients from the non-GCT control group often had tumour sites well known to be associated with thrombosis (inferior limb: 35%; pelvis: 14%). The duration of chemotherapy was comparable (Table 2).

### Thrombo-embolic events

Overall, 19 patients (19%) (95% confidence interval (CI): 13–28) and six patients (6%) (95% CI: 3–13) had a TEE in the GCT group and the non-GCT control group, respectively (relative risk (RR): 3.4; *P*<0.01). Initial TEE are described in Table 3. In most cases, the first TEE occurred during the course of primary chemotherapy. A second TEE occurred in eight and three patients in the GCT group and the non-GCT group, respectively (Table 4). Both patients who initially had a superficial venous thrombosis of the arm subsequently developed a deep venous thrombosis: thrombosis of the leg and pulmonary embolism in the first patient and lethal pulmonary embolism in the second patient. These two patients were only receiving a topical anti-inflammatory treatment for their superficial thrombosis when the deep venous thrombosis occurred. The other six patients were all receiving anticoagulant therapy when the second TEE occurred. Overall, two patients from the GCT group had an arterial TEE and both also had a venous TEE: in the first patient, a lethal pulmonary embolism occurred 6 weeks after a stroke, and in the second patient, a venous thrombosis of the superior cava vena related to a central catheter was followed 10 days later by an acute ischaemia of the leg (no long-term sequel after arterial thrombectomy).

Long-term complications occurred in six and three patients in the two groups, respectively. Three patients from the GCT group died of pulmonary embolism (one of them having also a stroke).

### Univariate analysis

A number of parameters were tested in univariate analysis to assess whether they were predictors of a first TEE in the GCT group (Table 5). Six parameters had a significant predictive value for TEE: age >30 years, a histology of pure seminoma, weight >70 kg, body surface area >1.9 m^2^, elevated serum lactate dehydrogenase (LDH), and elevated serum alkaline phosphatase.

### Multivariate analysis

Predictive factors of TEE identified in univariate analysis were tested in multivariate analysis using the Cox model. Two models were built by entering either weight or body surface area in the analysis. Results are summarised in Table 6. In the first model, two factors had independent predictive value for TEE: a high body surface area (>1.9 m^2^) and an elevated serum LDH. In the second model, weight over 70 kg and an elevated serum LDH were independent predictors of TEE.

The first model was used to build a risk index for TEE: patients with no risk factor (body surface area ⩽1.9 m^2^ and normal serum LDH; *n*=26) and those with at least one risk factor (*n*=71) had a probability of having a TEE of 4% (95% CI: 1–19) and 26% (95% CI: 17–37), respectively.

### Validation set

To validate these data, a separate set of 70 patients with GCT treated at the Centre Léon Bérard, Lyon, France, during the same period of time was analysed. Their characteristics are summarised in Tables 1 and 2. Patient characteristics were similar in the study set and the validation set. Overall, 10 (13%) of patients from the validation set had a TEE in the first 6 months following the start of chemotherapy (Tables 3 and 4). The only patient with a superficial venous thrombosis subsequently developed a deep venous thrombosis (leg and inferior vena cava).

Independent predictive factors of TEE identified in the study set were tested in this validation population: elevated serum LDH and a high body surface area were associated with a relative risk of TEE of 4.6 (95% CI: 0.6–37.9) and 1.7 (95% CI: 0.4–7.1) respectively. When the risk index of TEE was tested in this population, patients with no risk factor (body surface area ⩽1.9 m^2^ and a normal serum LDH; *n*=16) and those with at least one risk factor (*n*=60) had a probability of having a TEE of 0 and 17% (95% CI: 10–29), respectively.

## DISCUSSION

In this study, we provide evidence for the first time that patients with GCT who receive cisplatin-based chemotherapy are at a significantly higher risk of having a thrombosis in the subsequent 6 months compared with those with other cancers who received similar treatments. Moreover, the risk of having a thrombosis could be predicted in GCT patients using two simple factors: a high body surface area (>1.9 m^2^) (or a high weight) and an elevated serum LDH before chemotherapy. Patients with no risk factor had a risk of thrombosis of 4% while those with at least one risk factor had a risk of 26%. These features were 0 and 17% respectively in a validation set of patients with GCT treated in a different institution. Morbidity and mortality were relevant in this population of young men who are likely to achieve a cure of their cancer. TEE consisted mainly in venous thrombosis, with arterial events being more rare.

A large number of TEE occurring in GCT patients have been published as case reports (Doehn *et al*, 2000; Koga *et al*, 2001; Blokh *et al*, 2003; Leslie *et al*, 2003; cases published before 2000 reviewed in Weijl *et al*, 2000). In a British series of 333 patients with advanced GCT, the incidence of vena cava compression was 9.3%, of whom 29% had a thrombo-embolic complication and one patient died of pulmonary embolism (Hassan *et al*, 1999). Two previous studies also underscored the apparent high risk of TEE in GCT patients receiving chemotherapy, although there was no control group in these studies (Cantwell *et al*, 1988; Weijl *et al*, 2000). In a series of 52 patients, Cantwell *et al* (1988) reported 10 TEE (19%), including seven venous events, three arterial events, and one death. In another series of 179 patients, Weijl *et al* (2000) reported 15 TEE (8%), including 13 venous events, two arterial events, and one death. Moreover, a recent study with a 10-year follow-up showed that GCT survivors are at a higher cardiac risk than controls in the long term (Huddart *et al*, 2003). Therefore, based on our experience and previous publication, there is evidence that GCT patients have a three-fold higher risk of TEE (consisting mostly in venous events) during chemotherapy and soon afterwards, and that they have a two-fold higher risk of cardiac events in the long term.

The pathogenesis of TEE in GCT patients is not fully understood. Whether the causes are cancer related, treatment related, or both is still unknown. One weakness of our study was the relatively high number of parameters that were tested in univariate analysis compared to the limited number of patients (100). In the Weijl series, liver metastases and high doses of steroids (used as anti-emetics) were independent predictors of TEE, but these factors were not tested in a separate set of GCT patients (Weijl *et al*, 2000). In our study, the fact that CGT patients had a much higher risk for TEE than non-GCT cancer patients, even though both received cisplatin-based chemotherapy, indicates that cisplatin itself is not the only risk factor for TEE, in contrast with previous hypothesis (Icli *et al*, 1993). However, we can certainly not rule out a direct or indirect role of chemotherapy in the pathogenesis of TEE. Other drugs, in particular bleomycin (Schwarzer *et al*, 1991) and etoposide (Schwarzer *et al*, 1991; Airey *et al*, 1995), as well as the traditional 5-day protocols used in GCT might favour the occurrence of TEE, either via immobilisation or the daily use of steroids. A high body surface area (or weight) was identified as a risk factor of TEE in our study. This may be due to a direct effect since obesity is a well-known risk factor of venous TEE (Rosendaal, 1999). Alternatively, it may be due to an indirect factor related to chemotherapy since doses of chemotherapy drugs (except for bleomycin) are prescribed according to body surface area. Interestingly, hypercholesterolaemia has been suggested to be found more often in GCT survivors (Gietema *et al*, 1992; Raghavan *et al*, 1992; Meinardi *et al*, 2000) and this may be a potential explanation for arterial TEE during chemotherapy for GCT. In contrast, a recent study did not find any correlation between cisplatin administration and lipid profile in GCT survivors (Fenton *et al*, 2002).

Apart from the potential involvement of treatment-related factors, there is some evidence to support a direct contribution of GCT to the occurrence of TEE. For example, Weijl *et al* (2000) reported that a number of GCT patients experienced a TEE even though they had not received anticancer treatment. Indeed, we have made similar observations. Moreover, human teratocarcinoma cell lines release plasma membrane vesicles with procoagulant properties (Dvorak *et al*, 1983). Serum LDH is linked with tumour bulk and prognosis in advanced GCT (Fossa *et al*, 1997; IGCCCG, 1997) and we show in this study that this factor also predicts the risk of TEE. These findings indirectly support the role of GCT in the occurrence of TEE. Whether the secretion of LDH by GCT cells has a direct effect or is associated with the production of procoagulant factors is unknown.

In our opinion, the high incidence of TEE in GCT patients with either a high body surface area, a high serum LDH, or both, and who receive cisplatin-based chemotherapy is sufficient justification to prospectively evaluate the potential impact of thromboprophylaxis in these patients. However, this strategy needs to be cautiously balanced with the potential risk of thrombocytopenia (although usually mild) related to chemotherapy in patients with GCT. Recently, large studies have demonstrated that subcutaneous low-molecular-weight heparin can be safely used and reduces the risk of TEE in patients with acute medical illnesses (Samama *et al*, 1999). Moreover, recent randomised trials have suggested that low-molecular-weight heparin may be safer than vitamin K antagonists and that they may also be better in reducing the risk of TEE recurrence in cancer patients (Meyer *et al*, 2002; Lee *et al*, 2003, 2005).

## Figures and Tables

**Table 1:** Patients characteristics

	**GCT: study set (*n*=100)**	**GCT: validation set (*n*=77)**	**Non-GCT neoplasms: control group (*n*=100)**
Median age (range) (years)	30 (16–63)	32 (18–62)	34 (16–63)
Median weight (range) (kg)	73 (47–99)	72 (54–99)	71 (40–99)
			
*Performance status*			
0	76 (76%)	65 (85%)	78 (78%)
1	17 (17%)	9 (12%)	13 (13%)
2	6 (6%)	2 (3%)	8 (8%)
3	1 (1%)	0	1 (1%)
			
Smoking history	41/65 (63%)	32/67 (48%)	37/48 (77%)
			
*Histology and tumour site*			
GCT	86 (86%)	60 (78%)	
NSGCT	14 (14%)	17 (22%)	
Pure seminoma	86 (86%)	73 (94%)	
Testis	14 (14%)	4 (6%)	
Extragonadal			
Non-GCT			
Osteosarcoma			27
Lung cancer			16
Gastrointestinal tract cancer			14
Ewing's sarcoma			13
Head and neck cancer			12
Others			18
			
*Stage*			
Stage I	18 (18%)	7 (9%)	Localised: 43 (43%)
Stage II	42 (42%)	37 (48%)	Metastases: 57 (57%)
Stage III	40 (40%)	33 (43%)	
			
Median maximal tumour diameter (range) (mm)	43 (10–180)	30 (0–150)	68 (4–200)
Prophylactic anticoagulant therapy	12 (12%)	8/76 (11%)	21/96 (22%)
			
*Biology*			
Abnormal PT	5 (5%)	7/62 (11%)	5 (5%)
Abnormal APTT	5 (5%)	45/72 (63%)	5 (5%)
Elevated fibrinogen	17/22 (77%)		15/24 (63%)
Elevated LDH	41/97 (42%)		20/59 (34%)

GCT=germ-cell tumours; NSGCT=nonseminous germ-cell tumours; LDH=lactate dehydrogenase; PT=prothrombine time; APTT=activated partial thromboplastin time.

**Table 2:** Chemotherapy

**Chemotherapy**	**GCT: study group (*n*=100)**	**GCT: validation group (*n*=77)**	**Non-GCT neoplasms: control group (*n*=100)**
*Regimen*			
BEP	46 (46%)	46 (60%)	
EP	42 (42%)	28 (36%)	
CISCA-VB	12 (12%)	3 (4%)	
API-AI			31 (31%)
			
*Drugs*			
Cisplatin	100 (100%)	77 (100%)	100 (100%)
Etoposide	88 (88%)	74 (96%)	11 (11%)
Bleomycin	58 (58%)	49 (63%)	4 (4%)
Doxorubicin	12 (12%)	3 (4%)	31 (31%)
Cyclophosphamide	12 (12%)	3 (4%)	1 (1%)
Vinblastine	12 (12%)	3 (4%)	5 (5%)
Ifosfamide			36 (36%)
Epirubicine			11 (11%)
Vinorelbine			8 (8%)
5-Fluorouracil			7 (7%)
*α*-Interferon			2 (2%)
Thiotepa			1 (1%)
Docetaxel			1 (1%)
Interleukin-2			1 (1%)
Mitomycin C			1 (1%)
			
Duration of chemotherapy (days)	63 (6–408)	68 (4–129)	51 (3–258)
Neutropenic fever	19 (19%)	24 (33%)	16 (16%)
Postchemotherapy surgery	39 (39%)	40 (52%)	39 (39%)

CGT=germ-cell tumours.

**Table 3:** First TEE

	**GCT: study group (*n*=100)**	**GCT: validation group (*n*=77)**	**Non-GCT neoplasms: control group (*n*=100)**
Patients with a first TEE	19	10	6
			
*Timing of the first TEE*			
During chemotherapy	14	7	4
After the end of chemotherapy	5	3	2
			
*Deep venous thrombosis*	16	9	6
Leg	6	3	
Arm	4	1	
Inferior vena cava	4	1	5
Superior vena cava		1	1
Pulmonary embolism	1	2	
Renal vein	1	1	
Superficial venous thrombosis	2^a^	1^a^	0
			
*Arterial thrombosis*	1	0	0
Stroke	1		

a All these patients subsequently developed a deep venous thrombosis.

GCT=germ-cell tumours; TEE=thrombo-embolic events; PT=prothrombine time; .APTT=activated partial thromboplastin time.

**Table 4:** Complications of TEE

	**GCT: study group (*n*=19 first TEE)**	**GCT: validation group (*n*=10 first TEE)**	**Non-GCT neoplasms: control group (*n*=6 first TEE)**
Relapse of TEE	8^a^	1^a^	3^a^
			
*Venous*			
Pulmonary embolism	7		2
Leg	1	1	1
Arm	2		
Inferior vena cava		1	
			
*Arterial*			
Acute ischaemia of the leg	1		
			
*Long-term sequels*	6^b^	3^b^	3^b^
Oedema of the leg	4	2	2
Peripheral venous circulation or dermitis	2	1	
Chronic pain	2		
Walking limitation	1		
Renal failure			1
			
Death	3	0	0

a Some patients had multiple sites of second TEE.

b Some patients had multiple sequels.

TEE=thrombo-embolic events; GCT=germ-cell tumours.

**Table 5:** Univariate analysis of predictive factors for TEE in patients with GCT

**Characteristics**	**Number of TEE**	***P***	**Relative risk**
*Age (years)*			
⩽30	3	0.001	1
>30	16		6.8
			
*Weight (kg)*			
⩽70	3	0.002	1
>70	16		5.5
			
*Body surface area (m^2^)*			
⩽1.9	5	0.01	1
>1.9	14		3.4
			
			
*Performance status (WHO)*			
0–1	17	0.31	
2–3	2		
			
*Histology*			
Pure seminoma	6	0.01	3.4
NSGCT	13		1
			
*Primary tumour site*			
Testis	16	0.7	
Extragonadal	3		
			
*IGCCCG classification*			
Good	10	0.36	
Intermediate or poor	7		
			
*Stage before chemotherapy*			
Stage I	2	0.63	
Stage II	9		
Stage III	8		
			
*Maximal tumour diameter (mm)*			
⩽40	4	0.15	
>40	8		
			
*Central venous catheter*			
Yes	11	0.14	
No	8		
			
*Febrile neutropenia*			
Yes	6	0.09	2.2
No	13		1
			
*Preventive anticoagulant therapy*			
Yes	4	0.15	
No	15		
			
*Alkaline phosphatase*			
Normal	16	0.00001	1
Elevated	3		14.5
			
*PT, APTT*			
Norma	13	0.08	1
Abnormal	6		2.3
			
*LDH*			
Normal	5	0.01	1
Elevated	14		4.6

NSGCT=nonseminous germ-cell tumours; TEE=thrombo-embolic events; LDH=lactate dehydrogenase; PT=prothrombine time; APTT=activated partial thromboplastin time.

**Table 6:** Multivariate analysis

	**Relative risk (95% CI)**	***P***
*Model 1*		
Body surface area >1.9 m^2^	5.0 (1.8–13.9)	0.01
Elevated LDH	6.4 (2.3–18.2)	0.001
		
*Model 2*		
Weight >70 kg	7.4 (2.1–25.6)	0.01
Elevated LDH	5.9 (2.1–16.6)	0.01

LDH=lactate dehydrogenase.
